# Analysis of Amino Acids in the Roots of *Tamarix ramosissima* by Application of Exogenous Potassium (K^+^) under NaCl Stress

**DOI:** 10.3390/ijms23169331

**Published:** 2022-08-19

**Authors:** Yahui Chen, Shiyang Zhang, Shanfeng Du, Xiaomian Zhang, Jiang Jiang, Guangyu Wang

**Affiliations:** 1 Collaborative Innovation Center of Sustainable Forestry in Southern China of Jiangsu Province, Nanjing Forestry University, Nanjing 210037, China; 2 Department of Forest Resources Management and Faculty of Science, University of British Columbia, Vancouver, BC V6T 1Z4, Canada; 3 Zhejiang Academy of Forestry, Hangzhou 310023, China

**Keywords:** NaCl stress, exogenous potassium, salt poison, *T. ramosissima*, proline, amino acid

## Abstract

Soil salinization is one of the main environmental factors affecting plant growth worldwide. *Tamarix ramosissima* Ledeb. (*T. ramosissima*) is a halophyte representative that is widely grown in salinized soils. As an important nutrient element for plant growth, K^+^ plays an important role in improving the tolerance to salt stress, but the mechanism of reducing the damage caused by NaCl stress to *T. ramosissima* is less reported. Our results show that the proline content and the Log_2_ fold-change of proline’s relative quantification in the roots of *T. ramosissima* increased over time with the application of exogenous potassium (K^+^) for 48 h and 168 h under NaCl stress. Moreover, 13 amino-acid-related metabolic pathways were involved in the resistance of *T. ramosissima* to salt stress. Mainly, the aldehyde dehydrogenase family genes and tryptophan-synthase-related genes were found at 48 h and 168 h with exogenous potassium applied to the roots of *T. ramosissima* under NaCl stress, and they regulated their related metabolic accumulation in the arginine and proline metabolism pathways, increasing the effectiveness of inducing NaCl tolerance of *T. ramosissima*. It is noteworthy that alpha-ketobutyric was produced in the roots of *T. ramosissima* under NaCl stress for 48 h with the application of exogenous potassium, which is one of the most effective mechanisms for inducing salt tolerance in plants. Meanwhile, we found three DEGs regulating alpha-ketobutyric acid. This study provides a scientific theoretical basis for further understanding the molecular mechanism of K^+^ alleviating the salinity damage to *T. ramosissima* caused by NaCl.

## 1. Introduction

More than 6% of the world’s land area and approximately 20% of irrigated land is increasingly affected by salt accumulation [1,2]. Excessive salt accumulation harms plant growth and development, and even leads to plant death, resulting in agricultural yield losses and severe deterioration of plant ecosystems [1,3,4]. Therefore, it is necessary to improve the salt tolerance of plants.

Amino acids are important compounds in plants, playing an important role in regulating their growth processes. When encountering adversity stress, plants reduce the harm caused by adversity stress by controlling the absorption, synthesis and degradation of amino acids [5,6]. Therefore, accumulation and metabolism of amino acids are also crucial adaptive responses to abiotic stress in plants [7]. In recent years, research on improving plant stress resistance via amino acids has received extensive attention. Studies have shown that amino acids and some polyamines synthesized with amino acids as precursors can improve the adaptability of plants to salt stress [8,9,10,11]. Proline and aminobutyric acid have been studied more, and some progress has been made in elucidating their mechanisms of action. Proline, acting as an osmoregulatory compound, exists in plant cells as a free amino acid [12], and accumulates in large quantities under drought, salinity, and other stresses [13]; it can not only act as an osmoprotectant against the osmotic stress caused by salt stress, but also can scavenge reactive oxygen species (ROS) to protect plants against salt [14,15]. It has been shown that the exogenous application of proline reduces Na+ absorption in rice [16]. At the same time, proline can also regulate antioxidant enzyme activity and photosynthesis to improve the salt resistance of plants, promoting plant growth [17]. The increase in proline content can increase the Ca^2+^, K^+^, and Mg^2+^ plasma contents in various organs of plants under salt stress, reduce the Na^+^ and Cl^−^ contents, enhance the photosynthesis of plants—thereby promoting growth—and reduce the damage to plants caused by salinity stress. It can also enhance the ornithine synthesis of plants under salt stress, but has a certain inhibitory effect on glutamate synthesis, further enhancing the salinity stress tolerance of plants. Therefore, proline is a protective mechanism of plants against salt stress [18,19,20].

K^+^ is the most abundant monovalent cation in advanced plants, and it is essential for plants’ nutrition, growth, enzyme homeostasis, and osmotic pressure regulation [21]. Excessive accumulation of salt in plants can lead to excessive entry of Na^+^/Cl^−^ into the plant body, producing ion toxicity, and a high concentration of Na^+^ can inhibit K^+^ absorption in plants, cause K^+^ loss in roots and leaves, break the ion balance, and reduce the K^+^ uptake in plants [22,23,24]. However, Na^+^ is not necessary for plant growth, but K^+^ is necessary for the maintenance of various enzymes. Therefore, overaccumulation of Na^+^ could cause membrane damage, nutrient imbalance, altered growth regulator levels, enzyme inhibition, and metabolic disorders, preventing plants from growing normally [1,25]. Research has found that adding 10 mM exogenous KCl can effectively alleviate the toxic effect of drought stress on the growth of *Alternanthera philoxeroides* (Mart.) Griseb. and enhance the level of K^+^ enrichment in plants [26].

Due to their unique morphological structure, physiological function, and ecological characteristics, halophytes are highly adaptable to saline habitats [27]. *Tamarix* plants are typical representatives of halophytes, which are highly tolerant to various abiotic stresses—such as salt, drought, and high temperatures—during their long-term survival and evolution. They have developed efficient abiotic stress tolerance systems to adapt to adverse environments [28]. Studies have reported that *Tamarix ramosissima* Ledeb. (*T*. *ramosissima*) promotes its growth at low concentrations (≤100 mM NaCl) of salt stress and suppresses its growth at high concentrations (≥200 mM NaCl) of salt stress [29]. In this study, we investigated the candidate genes and metabolites of *T. ramosissima* in response to NaCl stress under the application of exogenous potassium at the physiological and molecular levels, and explored their amino-acid-related metabolic pathways involved in the response to NaCl stress. Our results provide a scientific theoretical basis for studying the molecular salinity tolerance mechanism of *T. ramosissima* plants under NaCl stress, variety breeding, and the NaCl stress alleviation effect of K^+^.

## 2. Results

### 2.1. Effect of Exogenous Potassium Application on the Proline Content of T. ramosissima Roots under NaCl Stress

Proline is a free amino acid. It can act as an osmoprotectant against osmotic stress caused by salt stress, and can also enhance the enzyme activity of the antioxidant defense system. In the present study (Supplementary Figure S1), the proline content in the control group did not change significantly within 168 h, while the proline content in the treatment groups increased over time. Among them, the proline content in the 200 mM NaCl + 10 mM KCl group increased the most. At 48 h, the proline content of the 200 mM NaCl + 10 mM KCl group was significantly increased compared to the control group. In contrast, the proline content of the 200 mM NaCl group was slightly increased compared to the control group, with no significant difference. At 168 h, the proline content of both treatment groups was significantly increased compared with the control group, and the proline content of the 200 mM NaCl + 10 mM KCl group was higher than that of the 200 mM NaCl group.

### 2.2. Analysis of the Log_2_ Fold-Change of the Proline’s Relative Quantification

The Log_2_ fold-change of proline’s relative quantification in the control group was the smallest within 168 h, while the Log_2_ fold-change of the 200 mM NaCl + 10 mM KCl and 200 mM NaCl groups increased over time. The Log_2_ fold-change of proline’s relative quantification in *T. ramosissima* roots showed a slowly increasing trend.

**Figure 1:**
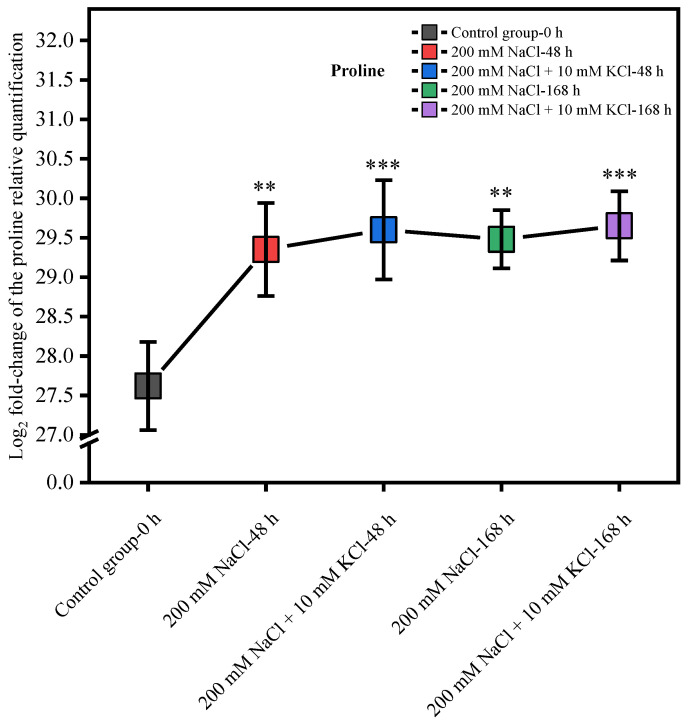
Log_2_ fold-change of proline’s relative quantification in the *T. ramosissima* roots under different treatments (Log_2_ fold-change of proline’s relative quantification in the roots of *T. ramosissima* under 200 mM NaCl + 10 mM KCl and 200 mM NaCl treatments. Note: *p* ≥ 0.05 is not marked; ** 0.001 < *p* < 0.01; *** *p* ≤ 0.001).

Among the treatment groups, at 48 h and 168 h, the Log_2_ fold-change of proline’s relative quantification in the roots of *T. ramosissima* in the 200 mM NaCl + 10 mM KCl group was greater than that in the 200 mM NaCl group. Moreover, the results of this study showed that the relative quantitative Log_2_ fold-change of proline in the 200 mM NaCl group and 200 mM NaCl + 10 mM KCl group increased relative to the control group, and the difference was significant (Figure 1).

### 2.3. Analysis of Metabolic Pathways Associated with Amino Acids in T. ramosissima Roots under NaCl Stress

Thirteen amino-acid-related metabolic pathways (the cysteine and methionine metabolism pathway; tryptophan metabolism pathway; arginine and proline metabolism pathway; phenylalanine metabolism pathway; histidine metabolism pathway; tyrosine metabolism pathway; alanine metabolism pathway; aspartate and glutamate metabolism pathway; glycine, serine, and threonine metabolism pathway; valine, leucine, and isoleucine biosynthesis pathway; arginine biosynthesis pathway; phenylalanine, tyrosine, and tryptophan biosynthesis pathway; lysine biosynthesis pathway; and biosynthesis of amino acids pathway) were discovered in the roots of *T. ramosissima* when treated with exogenous potassium for 48 h and 168 h under NaCl stress.

In the 200 mM NaCl 48 h vs. 200 mM NaCl + 10 mM KCl 48 h comparison group, 13 metabolic pathways involved 14 differential metabolites—11 upregulated and 3 downregulated—and the metabolic pathways also involved 40 DEGs, 14 of which were upregulated and 26 of which were downregulated (Supplementary Table S1). In the 200 mM NaCl 168 h vs. 200 mM NaCl + 10 mM KCl 168 h comparison group, 12 metabolic pathways involved 19 differential metabolites—11 upregulated and 8 downregulated—and the metabolic pathways also involved 57 DEGs, 12 of which were upregulated and 45 of which were downregulated (Supplementary Table S2). A total of seven differential metabolites was included in the 200 mM NaCl 48 h vs. 200 mM NaCl + 10 mM KCl 48 h and 200 mM NaCl 168 h vs. 200 mM NaCl + 10 mM KCl 168 h comparison groups—(S)-alpha-amino-beta-(3-indolyl)-propionic acid, 5-aminovaleric acid, 4-hydroxy-3-methoxybenzaldehyde, 2,6-diaminohexanoic acid, N2-Acetyl-L-ornithine, 2-coumarate, and 3-methoxytyramine—along with four DEGs (*Unigene0004792*, *Unigene0019527*, *Unigene0050969*, and *Unigene0088939*).

### 2.4. Analysis of the Arginine and Proline Metabolic Pathway in T. ramosissima Roots with Exogenous Potassium Applied under NaCl Stress

**Figure 2:**
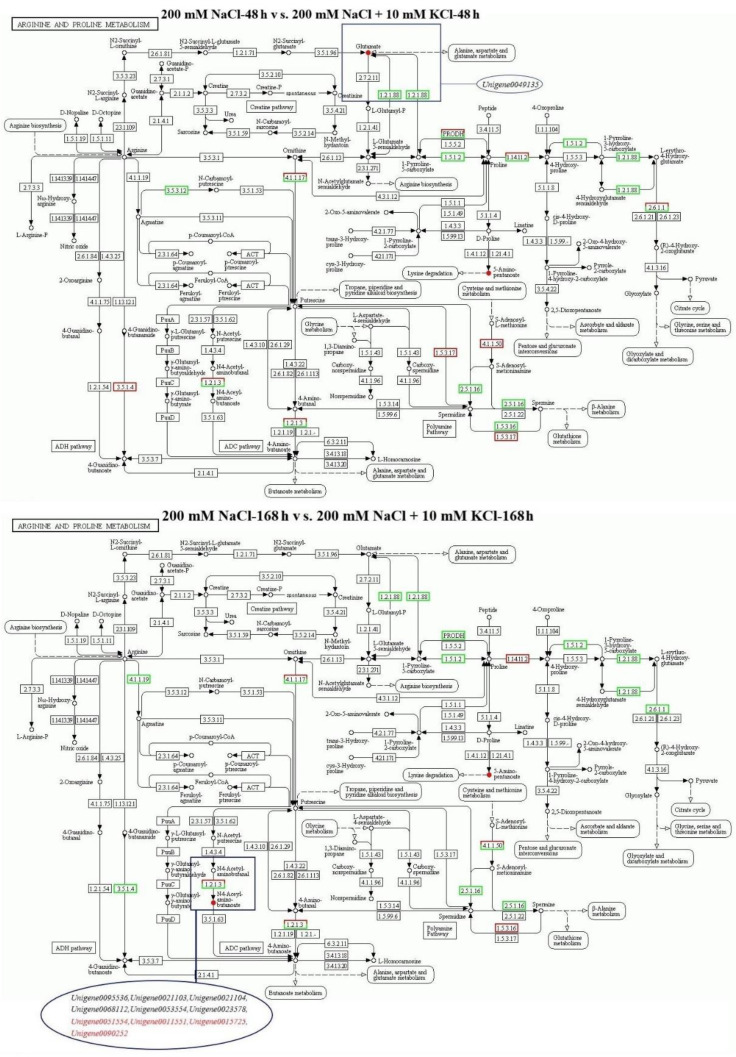
Analysis of the arginine and proline metabolic pathway: The changes in the annotated expression levels of DEGs and differential metabolites in the arginine and proline metabolic pathway, and the regulatory relationship between DEGs and differential metabolites in the 200 mM NaCl 48 h vs. 200 mM NaCl + 10 mM KCl 48 h and 200 mM NaCl 168 h vs. 200 mM NaCl + 10 mM KCl 168 h comparison groups. Note—Black: gene expression level is downregulated, metabolites are degraded; red: gene expression level is upregulated, metabolites are accumulated.

Based on transcriptomic and metabolomic data (Supplementary Table S3), we performed an association analysis of the arginine and proline metabolic pathway (Table 1), and the results showed that there were two differential metabolites (glutamate and 5-aminopentanoate) accumulated in the 200 mM NaCl 48 h vs. 200 mM NaCl + 10 mM KCl 48 h comparison group (Supplementary Table S4). Among them, the expression level of *Unigene0049135* was downregulated, and appeared both upstream and downstream of glutamate, negatively regulating the accumulation of glutamate (Figure 2). There were two differential metabolites (N4-acetylaminobutanoate and 5-aminopentanoate) accumulated in the 200 mM NaCl 168 h vs. 200 mM NaCl + 10 mM KCl 168 h comparison group (Supplementary Table S4). Among them, six DEGs (*Unigene0095536*, *Unigene0021103*, *Unigene0021104*, *Unigene0068112*, *Unigene0053554*, and *Unigene0023578*) were downregulated, and appeared upstream of N4-acetylaminobutanoate, negatively regulating the accumulation of N4-acetylaminobutanoate. Moreover, four DEGs (*Unigene0051554*, *Unigene0011551*, *Unigene0015725*, and *Unigene0090252*) appeared to be upregulated and located upstream of N4-acetylaminobutanoate, positively regulating N4-acetylaminobutanoate accumulation.

**Figure 3:**
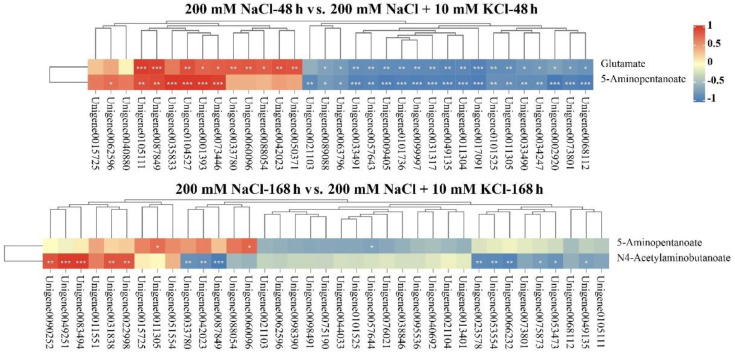
Pearson’s correlation clustering heatmap analysis of DEGs and differential metabolites in the arginine and proline metabolic pathway: The DEGs and differential metabolites contained in Pearson’s correlation clustering heatmap analysis based on the 200 mM NaCl 48 h vs. 200 mM NaCl + 10 mM KCl 48 h and 200 mM NaCl 168 h vs. 200 mM NaCl + 10 mM KCl 168 h comparison groups in the arginine and proline metabolic pathway. Note: *p* ≥ 0.05 is not marked; * 0.01 < *p* < 0.05; ** 0.001 < *p* < 0.01; *** *p* ≤ 0.001.

In addition, in the 200 mM NaCl 48 h vs. 200 mM NaCl + 10 mM KCl 48 h comparison group, 33 DEGs and 2 differential metabolites were involved in the arginine and proline metabolism pathway (Supplementary Table S5). In the 200 mM NaCl 168 h vs. 200 mM NaCl + 10 mM KCl 168 h comparison group, 37 DEGs and 2 differential metabolites were involved in the arginine and proline metabolism pathway (Supplementary Table S5). We performed Pearson’s correlation thermal cluster map analysis of the DEGs and differential metabolites listed in Supplementary Table S5 (Figure 3). The results showed that there were 28 DEGs significantly correlated with glutamate and 26 DEGs significantly correlated with 5-aminopentanoate in the 200 mM NaCl 48 h vs. 200 mM NaCl + 10 mM KCl 48 h comparison group. Notably, *Unigene0049135* was significantly negatively correlated with both glutamate and 5-aminopentanoate. Three DEGs were significantly correlated with 5-aminopentanoate and fourteen DEGs were significantly correlated with N4-acetylaminobutanoate in the 200 mM NaCl 168 h vs. 200 mM NaCl + 10 mM KCl 168 h comparison group. Among them, *Unigene0053554* and *Unigene0023578* were significantly negatively correlated with N4-acetylaminobutanoate. It is noteworthy that *Unigene0090252* was significantly positively correlated with N4-acetylaminobutanoate.

### 2.5. Phylogenetic Tree Analysis of Key Amino Acid Candidate Genes in T. ramosissima

According to the results shown in Section 2.4, the expression of *Unigene0090252* was upregulated in the comparison group of 200 mM NaCl 168 h vs. 200 mM NaCl + 10 mM KCl 168 h in the arginine and proline metabolic pathway, and the N4-acetylaminobutanoate was positively regulated, with a statistically significant difference. In particular, *Unigene0090252* was positive correlated with N4-acetylaminobutanoate, and the correlation was significant. As a key candidate gene, *Unigene0090252* showed an upward trend in its expression level within 168 h of exogenous potassium addition under NaCl stress (Supplementary Figure S2). This indicated that *Unigene0090252* had a long-term effect in enhancing the NaCl tolerance of *T. ramosissima* within 168 h of exogenous potassium addition under NaCl stress. Therefore, the protein amino acid sequence of *Unigene0090252* was selected and compared using BLAST from the National Center for Biotechnology Information (NCBI). Nineteen homologous gene species were selected in this study (Table 2). Using MEGA software, the phylogenetic tree was constructed by combining the protein amino acid sequence of *Unigene0090252* of *T. ramosissima* and the protein amino acid sequence of these 19 homologous gene species. The results showed that *Unigene0090252* was closely related to *Spinacia oleracea* (Supplementary Figure S3).

### 2.6. Quantitative Real-Time PCR (qRT-PCR) Validation of DEGs

**Figure 4:**
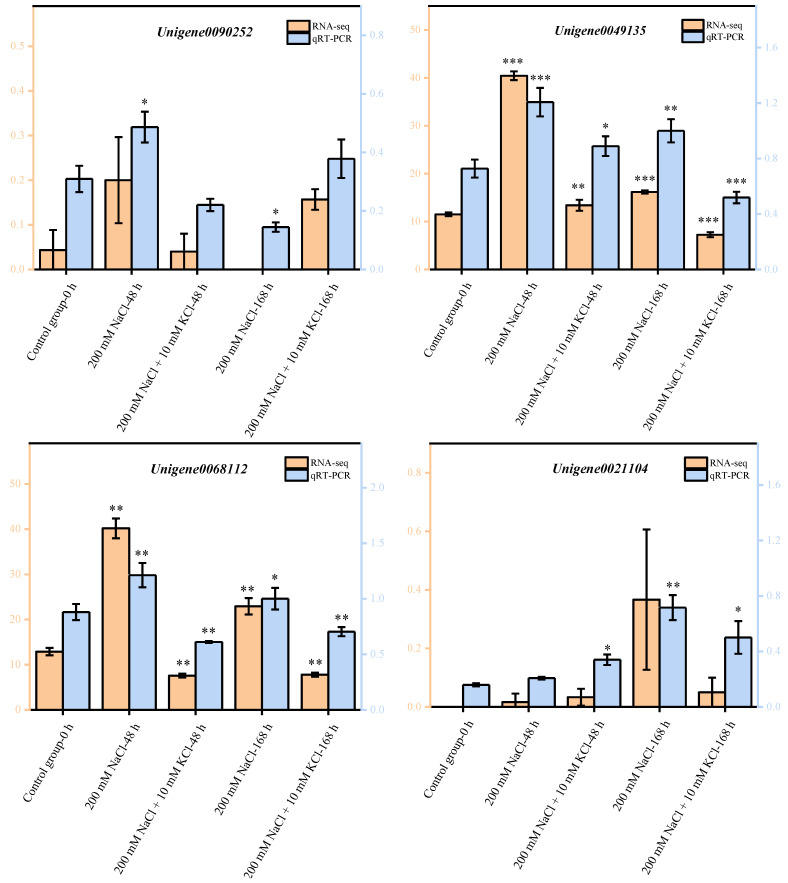

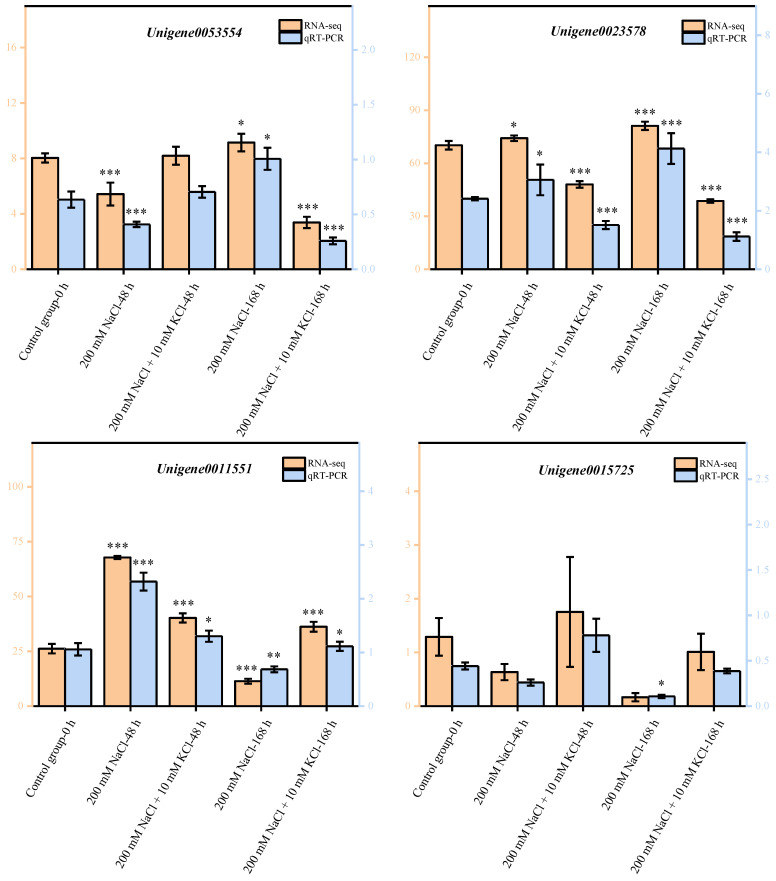
Validation of DEGs by qRT-PCR: Eight DEGs were randomly selected for qRT-PCR validation. The error bars were obtained from multiple replicates of qRT-PCR. Note: *p* ≥ 0.05 is not marked; * 0.01 < *p* < 0.05; ** 0.001 < *p* < 0.01; *** *p* ≤ 0.001. 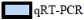: Numerical value is shown on the right side of the *Y* axis; 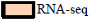: Numerical value is shown on the left side of the *Y* axis.

Eight DEGs were randomly selected and verified for qRT-PCR according to Chen’s method [30]. The results showed that the qRT-PCR validation results were entirely consistent with the expression trend of the transcriptome sequencing analysis results (Figure 4), proving that the transcriptomic data obtained in this study were accurate and reliable. This result can provide a theoretical basis for mining key candidate genes that could improve salt tolerance and alleviate NaCl stress damage in *T. ramosissima* roots.

## 3. Discussion

Salt stress affects almost all metabolic and developmental processes of plants [31], activating certain specific genes that maintain metabolic balance in plants, cause changes in plants, and eventually show regulatory results at the metabolite level. Especially when plants are subjected to abiotic stress, they undergo various changeable and complex physiological and biochemical reactions, whose response changes are regulated in multiple ways [32]. Transcriptomics studies the changes in response to transcriptional regulation after stress, while metabolomics analyses the changes in metabolic components and metabolic pathways after stress [33]. Through physiological, transcriptomics, and metabolomics association analyses, we can better understand the different times and treatments of the *T. ramosissima* roots’ physiology, the correlations of DEGs and differential metabolites, and the DEGs and differential metabolites involved in the metabolic pathways. It is essential to reveal the physiological and molecular mechanisms of NaCl tolerance in *T*. *ramosissima.*

As both an amino acid and an important osmoprotectant, proline accumulates significantly under salt stress, and is able to improve salt tolerance [34]. It plays a crucial role in stabilizing plant cell membranes [35], and the massive accumulation of proline is beneficial [36] for stressed plants. In this study, the proline content in the NaCl + KCl group and the NaCl group continuously increased under 48 h and 168 h of exogenous potassium application to *T. ramosissima*. The results showed that under NaCl stress, the accumulation of proline in the roots of *T. ramosissima* increased under the exogenous application of potassium, enabling better regulation of the intracellular osmotic potential, protecting the integrity of the cell structure, and improving the salt tolerance.

Several amino acid metabolic pathways related to salt stress were identified based on the transcription and metabolic data correlation analysis of *T. ramosissima* roots under NaCl stress. Importantly, arginine and proline positively affect membrane integrity, while also playing an adaptive role in regulating plants’ osmoregulation under stress conditions [37]. In this study, *Unigene0051554*, *Unigene0011551*, *Unigene0015725*, and *Unigene0090252*, belonging to the aldehyde dehydrogenase family, regulated upstream of the differential metabolite N4-acetylaminobutanoate in the arginine and proline metabolic pathways, and were positively correlated. Reports showed that aldehyde dehydrogenase can increase the antioxidant enzyme activity, root activity, and chlorophyll content of plants under NaCl stress, stabilize the permeability of cell membranes, and reduce the accumulation of Na^+^ in plants, thereby reducing the degree of salt damage to seedlings [38]. Therefore, in this study, some aldehyde dehydrogenase family genes (*Unigene0051554*, *Unigene0011551*, *Unigene0015725*, and *Unigene0090252*) were involved in improving the NaCl tolerance of *T. ramosissima* roots. The cysteine and methionine metabolic pathway is extremely important for the induction of alternative oxidase pathways in response to plant salinity stress [39]. Studies have reported that the production of alpha-ketobutyrate, which can reduce the ACC levels in plants and prevent the excessive increase in ethylene synthesis under various stress conditions, is one of the most effective mechanisms for inducing salt tolerance in plants [40]. This study showed that the roots of *T. ramosissima* were exposed to exogenous potassium under NaCl stress in the cysteine and methionine metabolic pathway; the glycine, serine, and threonine metabolic pathway; the valine, leucine, and isoleucine biosynthesis metabolic pathway; and the biosynthesis of amino acids metabolic pathway. The alpha-ketobutyrate of the biosynthesis pathway and the amino acid biosynthesis metabolic pathway showed upregulated trends at 48 h. *Unigene0037977* negatively regulated alpha-ketobutyrate upstream of the valine, leucine, and isoleucine biosynthesis metabolic pathway. *Unigene0037977* negatively regulated alpha-ketobutyrate downstream of the amino acid biosynthesis metabolic pathway. Meanwhile, three DEGs (*Unigene0032304*, *Unigene0062653*, and *Unigene0062655*) positively regulated alpha-ketobutyrate downstream of the amino acid biosynthesis metabolic pathway, indicating that these three DEGs (played an important role in regulating alpha-ketobutyrate to improve the NaCl tolerance of *T*. *ramosissima*.

L-tryptophan is considered to be a potent physiological precursor of auxin [41]. Its application alleviates the adverse effects of salinity [42]. Meanwhile, tryptophan synthase can synthesize important components of L-tryptophan. In this study, the L-tryptophan synthase genes (*Unigene0019527* and *Unigene0088939*) promoted L-tryptophan synthesis in the roots of *T. ramosissima* after 48 h and 168 h of exogenous potassium application under NaCl stress. These two genes also regulated the upstream and downstream of (S)-alpha-amino-beta-(3-indolyl)-propionic acid. However, (S)-alpha-amino-beta-(3-indolyl)-propionic acid remained upregulated at 48 h and 168 h. This may be related to their regulation, considering the effectiveness of tryptophan-induced salt resistance of *T. ramosissima.* Finally, the growth of *T. ramosissima* under salt stress conditions was consistent with the findings of Zahir et al. [43].

Finally, amino acids were involved in the application of exogenous potassium to improve the salt resistance of multiple *T. ramosissima* roots under NaCl stress, but the process that helps to alleviate NaCl toxicity is a more complex molecular mechanism.

## 4. Materials and Methods

### 4.1. Plant Materials

Five-month-old *T. ramosissima* seedlings with similar growth were selected and transferred to a 24-hole hydroponic box (40 cm × 30 cm × 16 cm in size) filled with 1/2 Hoagland nutrient solution. In a greenhouse at 26 ± 2 °C and with relative humidity of 40% to 55%, the seedlings were cultivated for 2 months before use. The experiment was conducted from October 2019 to May 2021 in the Key Laboratory of the Ministry of Education, Nanjing Forestry University.

### 4.2. Plant Materials’ Treatment

In the experiment, 1/2 Hoagland nutrient solution culture was used as a control group, compared with 1/2 Hoagland nutrient solution cultures supplemented with 200 mM NaCl and 200 mM NaCl + 10 mM KCl, and replaced once every 3 days. Control and treatment groups were set for each trial, with 8 plants in each group and 3 replicates per group. Finally, *T*. *ramosissima* root samples were collected after 0 h, 48 h, and 168 h of experimental treatment, and immediately placed in liquid nitrogen and transferred to a −80 °C refrigerator for storage.

### 4.3. Determination of Proline Content in the Roots of T. ramosissima under Different Treatments

*T. ramosissima* treated with the control, 200 mM NaCl, and 200 mM NaCl + 10 mM KCl for 48 h and 168 h were randomly selected, and their root tissues were sampled with three replicates. The proline contents were measured according to the method of Abrahám et al., and three biological replicates were performed for each determination [44].

### 4.4. Transcriptome Sequencing and Screening of DEGs

After liquid nitrogen treatment, according to the method of Chen et al. [45], *T. ramosissima* root samples were subjected to 3 generations of high-throughput transcriptome sequencing. The original Illumina sequencing data were then submitted to the National Center for Biotechnology Information (NCBI) Short Reads Archive (SRA) database (SRP356215). The reads count data obtained from sequencing were analyzed utilizing DESeq2 [46] to obtain the *p*-value after BH correction (FDR value), and a corrected *p*-value < 0.05 was considered statistically significantly enriched. According to the differential analysis results, we screened the genes with an FDR < 0.05 and a |log_2_FC| > 1 as significant DEGs. Finally, we used Gene Ontology (GO) [47] and the Kyoto Encyclopedia of Genes and Genomes (KEGG) [48] to perform enrichment analysis on the acquired DEGs.

### 4.5. Metabolic Extraction, Detection, and Differential Metabolite Screening

After treatment with liquid nitrogen, the *T. ramosissima* root samples were extracted and detected according to the method of Chen et al. [45], and then analyzed via liquid chromatography–mass spectrometry (LC–MS).

LC–MS analyses were performed using a Vanquish UHPLC system (Thermo Fisher, Bremen, Germany) coupled with an Orbitrap Q Exactive^TM^ HF-X mass spectrometer (Thermo Fisher, Bremen, Germany). Samples were injected onto a Hypersil Gold column (100 × 2.1 mm, 1.9 μm) using a 17-min linear gradient at a flow rate of 0.2 mL/min. The eluents for the positive polarity mode were eluent A (0.1% FA in water) and eluent B (methanol). The eluents for the negative polarity mode were eluent A (5 mM ammonium acetate, pH 9.0) and eluent B (methanol). The solvent gradient was set as follows: 2% B, 1.5 min; 2–100% B, 12.0 min; 100% B, 14.0 min; 100–2% B, 14.1 min; 2% B, 17 min. The Q Exactive^TM^ HF-X mass spectrometer was operated in positive/negative polarity mode with spray voltage of 3.2 kV, capillary temperature of 320 °C, sheath gas flow rate of 40 arb, and aux gas flow rate of 10 arb. Finally, we used Simca software [49] to perform log-transformations, centralize the format, and carry out orthogonal projections to latent structures discriminant analysis (OPLS-DA) for all normalized data. Differential metabolites were screened based on the VIP > 1.0 for the first principal component of the OPLS-DA model, and *p* < 0.05 for the *t*-test [50].

### 4.6. Phylogenetic Tree Construction of Key Candidate Genes

Based on the association analysis of transcriptomic and metabolomic data, key candidate genes were identified, and then their protein and amino acid sequences were selected for comparison using BLAST from the National Center for Biotechnology Information (NCBI). Finally, the phylogenetic tree was constructed from key candidate genes and protein amino acid sequences of various homologous gene species.

### 4.7. Quantitative Real-Time PCR (qRT-PCR) Validation of DEGs

Eight DEGs were randomly selected to verify the accuracy of the RNA-Seq results. Total RNA was extracted from *T*. *ramosissima* root samples using Omega Kit (Beinuo Bio, Shanghai, China) from Omega Bio-Tek. It was then reverse-transcribed into strand cDNA as a template using the PrimeScript^TM^ RT Master Mix (Perfect Real Time) kit from Bao Bioengineering, TaKaRa (Bao Bio, Dalian, China). We designed DEG-specific expression primers (Supplementary Table S6), and collected root samples using Thermo Fisher Technology’s PowerUp^TM^ SYBR Green Master Mix reagent (Thermo Fisher, Shanghai, China), The qRT-PCR tests were performed using the ABI ViiA™ 7 Real-Time PCR System (ABI, Carlsbad, CA, USA) instrument from Applied BioSystems, with 4 technical replicates and 3 biological replicates for each candidate gene. Relative expression was calculated by 2^−ΔΔCt^ [45], using tubulin as the reference gene.

### 4.8. Experimental Data Processing

Statistical analysis of the data was performed using Excel (Microsoft, Washington, DC, USA); significance analysis was performed using SPSS 26.0 (IBM, Chicago, IL, USA), graphs were drawn using Origin 2018 software (OriginLab Corporation, Northampton, MA, USA), and phylogenetic trees were created using MEGA 11 software (MEGA Software, Richlandtown, PA, USA). In this study, we used untargeted metabolomics and the results obtained by LC–MS analysis as relative quantitative values without units, and then used ANOVA for significance testing, and the proline content determination, transcriptome sequencing, and metabolite detection were each repeated 3 times technically and 3 times biologically.

## 5. Conclusions

The increased proline content as a result of applying exogenous potassium for 48 h and 168 h under NaCl stress in *T. ramosissima* roots eventually improved plants’ salt tolerance. The transcriptomic and metabolomic data association analysis results showed that 13 amino-acid-related metabolic pathways were involved in *T. ramosissima*’s resistance to NaCl stress. In particular, four aldehyde dehydrogenase family genes (*Unigene0051554*, *Unigene0011551*, *Unigene0015725*, and *Unigene0090252*) were found in 48 h and 168 h under NaCl stress; these genes positively regulated N4-acetylaminobutanoate upstream, improving the antioxidant enzyme activity and the roots’ vitality, stabilizing the permeability of the cell membranes, and reducing the accumulation of Na^+^ in plants, thus reducing the degree of salinity damage to *T. ramosissima*. In particular, *Unigene0090252* was positively correlated with N4-acetylaminobutanoate, and there was a significant difference between groups. Two tryptophan-synthase-related genes (*Unigene0019527* and *Unigene0088939*) were also found in 48 h and 168 h with exogenous potassium applied under NaCl stress. These genes can synthesize tryptophan, inducing salt resistance to increase the plants’ growth under salinity stress conditions, and *Unigene0019527* and *Unigene0088939* also positively regulate the upstream and downstream of (S)-alpha-amino-beta-(3-indolyl)-propionic acid. In addition, alpha-ketobutyric acid was produced in the roots of *T*. *ramosissima* under NaCl stress when exogenous potassium was applied for 48 h, reducing the levels of ACC in the plants, preventing the excessive increase in ethylene synthesis under various stress conditions, and inducing salt tolerance. This is currently one of the most effective mechanisms. In particular, three DEGs (*Unigene0032304*, *Unigene0062653*, and *Unigene0062655*) played an important role in regulating alpha-ketobutyrate to improve the NaCl tolerance of *T*. *ramosissima*.

In conclusion, amino acids such as proline, tryptophan, and glutamic acid participated in the process of exogenous potassium application under NaCl stress through various pathways to improve the NaCl tolerance of *T. ramosissima*. However, this process is a relatively complex molecular mechanism that merits further study. This study provides a scientific theoretical basis for further mining the key salt tolerance genes, metabolic pathways, and metabolites through which K^+^ alleviates NaCl stress.

## Figures and Tables

**Table 1:** Arginine and proline metabolic pathway.

Number	Pathway	Candidate Genes with Pathway Annotation	Gene *p*-Value	Candidate Metabolites with Pathway Annotation	Metabolite *p*-Value	Pathway ID
200 mM NaCl 48 h vs. 200 mM NaCl + 10 mM KCl 48 h						
24	Arginine and proline metabolism	33	0.757413	2	0.474945	ko00330
200 mM NaCl 168 h vs. 200 mM NaCl + 10 mM KCl 168 h						
7	Arginine and proline metabolism	37	0.112464	2	0.564616	ko00330

Note: 24 and 7 represent the KEGG pathway rankings.

**Table 2:** Information sheet for the 19 homologous gene species.

Family	Species	Description	Protein ID	CDS (bp)	ORF Length (aa)
Amaranthaceae	*Spinacia oleracea*	Aldehyde dehydrogenase family 3 member F1	XP_021849540.1	1467	488
Vitaceae	*Vitis riparia*	Aldehyde dehydrogenase family 3 member F1	XP_034682258.1	1488	495
Rosaceae	*Prunus avium*	Aldehyde dehydrogenase family 3 member F1	XP_021815051.1	1464	487
Cucurbitaceae	*Cucurbita maxima*	Aldehyde dehydrogenase family 3 member F1	XP_022984451.1	1434	477
Solanaceae	*Datura stramonium*	Aldehyde dehydrogenase 3 member F1	MCD7447503.1	1461	486
Rhamnaceae	*Ziziphus jujuba var. spinosa*	Aldehyde dehydrogenase family 3 member F1	XP_048335012.1	1455	484
Fagaceae	*Quercus suber*	Aldehyde dehydrogenase family 3 member F1-like	XP_023880319.1	1443	480
Rosaceae	*Prunus persica*	Aldehyde dehydrogenase family 3 member F1 isoform X2	XP_020423669.1	1503	500
Malvaceae	*Gossypium hirsutum*	Aldehyde dehydrogenase family 3 member F1 isoform X1	XP_016692426.1	1452	483
Rubiaceae	*Coffea eugenioides*	Aldehyde dehydrogenase family 3 member F1-like isoform X1	XP_027178566.1	1467	488
Celastraceae	*Tripterygium wilfordii*	Aldehyde dehydrogenase family 3 member F1 isoform X1	XP_038697178.1	1473	490
Fabaceae	*Senna tora*	Aldehyde dehydrogenase family 3 member F1	KAF7828920.1	1458	485
Myricaceae	*Morella rubra*	Aldehyde dehydrogenase family 3 member F1	KAB1204492.1	1443	480
Rubiaceae	*Coffea arabica*	Aldehyde dehydrogenase family 3 member F1 isoform X1	XP_027074002.1	1467	488
Myrtaceae	*Syzygium oleosum*	Aldehyde dehydrogenase family 3 member F1	XP_030469961.1	1443	480
Rosaceae	*Pyrus* × *bretschneideri*	Aldehyde dehydrogenase family 3 member F1	XP_009378224.2	1494	497
Solanaceae	*Solanum pennellii*	Aldehyde dehydrogenase family 3 member F1 isoform X1	XP_015066806.1	1470	489
Euphorbiaceae	*Jatropha curcas*	Aldehyde dehydrogenase family 3 member F1	XP_012078389.1	1449	482
Malvaceae	*Hibiscus syriacus*	Aldehyde dehydrogenase family 3 member F1	XP_038994791.1	1440	479

## Data Availability

Not applicable.

## Supplementary Materials

The following supporting information can be downloaded at: https://www.mdpi.com/article/10.3390/ijms23169331/s1.
