# Supplementary material for: Analysis of Amino Acids in the Roots of Tamarix ramosissima by Application of Exogenous Potassium (K+) under NaCl Stress
Source: Int J Mol Sci. 2022 Aug 19;23(16):9331. doi: 10.3390/ijms23169331 (PMC9409283; doi:10.3390/ijms23169331)
Supplement: Supplementary file 1 [file ijms-23-09331-s001.zip › Supplementary Figure S1.pdf]

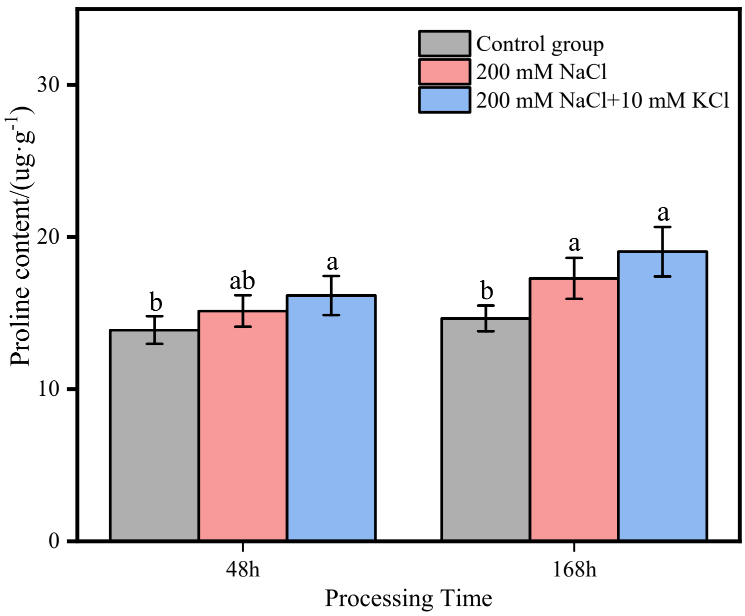

Supplementary Figure S1. Effects of exogenous potassium application on proline content in the roots of *T. ramosissima* under NaCl stress

(Changes of proline content in the roots of *T. ramosissima* at 48 h and 168 h under different treatments. Note： different letters simultaneously indicate significant differences among treatments, $p < 0.05$).
