# Supplementary material for: Analysis of Amino Acids in the Roots of Tamarix ramosissima by Application of Exogenous Potassium (K+) under NaCl Stress
Source: Int J Mol Sci. 2022 Aug 19;23(16):9331. doi: 10.3390/ijms23169331 (PMC9409283; doi:10.3390/ijms23169331)
Supplement: Supplementary file 1 [file ijms-23-09331-s001.zip › Supplementary Figure S2.pdf]

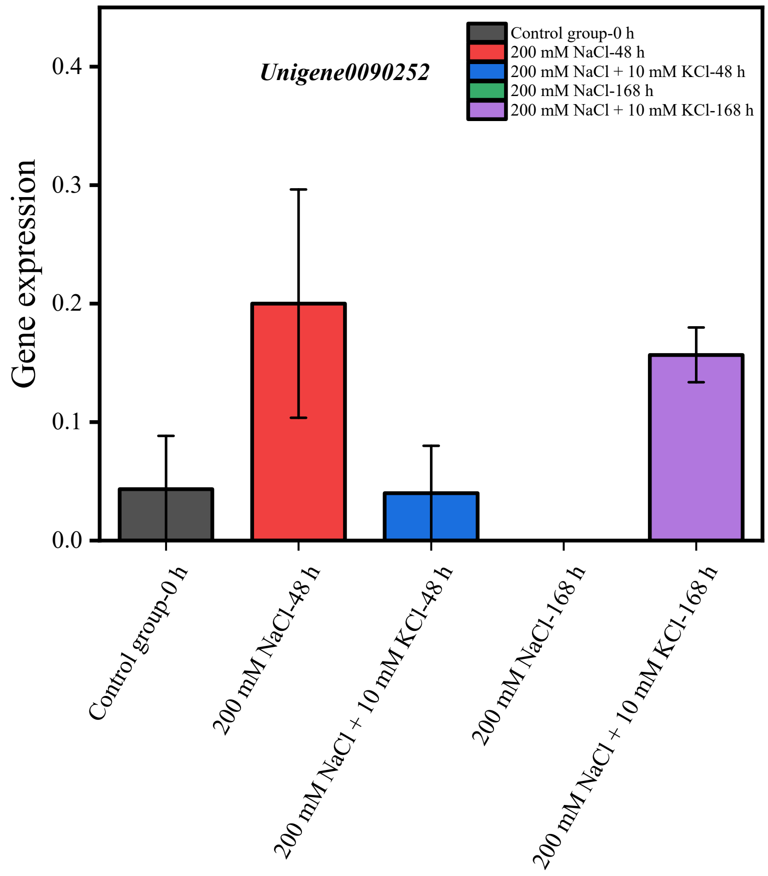

Supplementary Figure S2. Changes in the expression level of *Unigene0090252*

(Changes in the expression of *Unigene0090252* under NaCl stress at 48h and 168h with exogenous potassium applied).
