# Supplementary material for: Analysis of Amino Acids in the Roots of Tamarix ramosissima by Application of Exogenous Potassium (K+) under NaCl Stress
Source: Int J Mol Sci. 2022 Aug 19;23(16):9331. doi: 10.3390/ijms23169331 (PMC9409283; doi:10.3390/ijms23169331)
Supplement: Supplementary file 1 [file ijms-23-09331-s001.zip › Supplementary Figure S3.pdf]

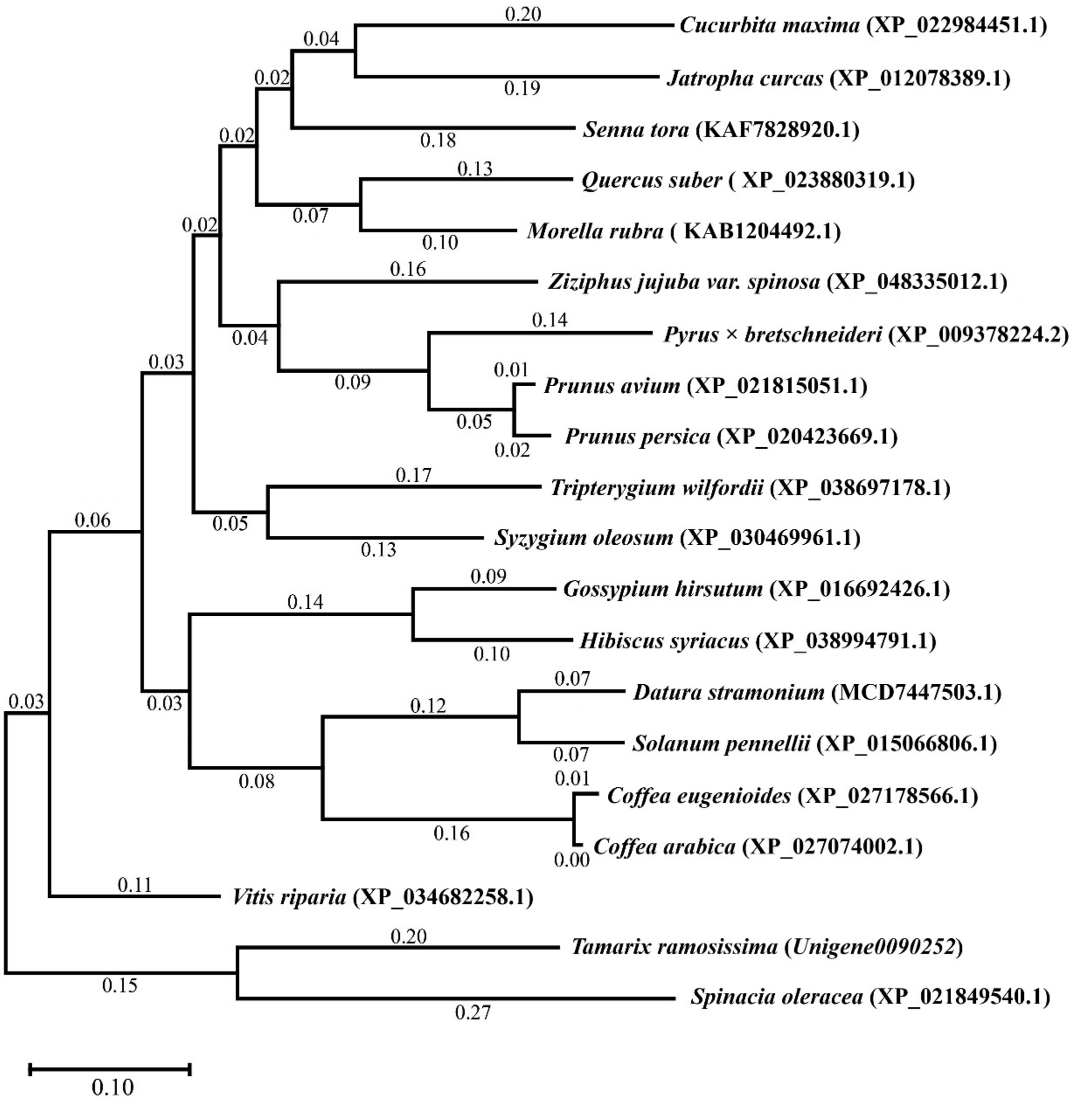

Supplementary Figure S3. Phylogenetic tree analysis of *T. ramosissima* amino acid and other species amino acid

(Phylogenetic tree analysis of Unigene0090252 protein amino acid sequence and protein amino acid sequence of other 19 species of *T. ramosissima*).
