# Supplementary material for: Analysis of Amino Acids in the Roots of Tamarix ramosissima by Application of Exogenous Potassium (K+) under NaCl Stress
Source: Int J Mol Sci. 2022 Aug 19;23(16):9331. doi: 10.3390/ijms23169331 (PMC9409283; doi:10.3390/ijms23169331)
Supplement: Supplementary file 1 [file ijms-23-09331-s001.zip › Supplementary Table S1.pdf]

Supplementary Table S1. Analysis of differential metabolites and DEGs in 200 mM NaCl 48 h vs. 200 mM NaCl+10 mM KCl 48 h amino acid-related pathways

Pathway	200 mM NaCl 48 h vs. 200 mM NaCl+10 mM KCl 48 h		
	Metabolites	Genes	Regulation
Cysteine and methionine metabolism	**alpha-Ketobutyrate**		
Tryptophan metabolism	**(S)-alpha-Amino-beta-(3-indolyl)-propionic acid**	***Unigene0010455***，*Unigene0038716*，***Unigene0037143***	down
Arginine and proline metabolism	**Glutamate**	*Unigene0049135*	up & down
	**5-Aminovaleric acid**		
Phenylalanine metabolism	**(S)-alpha-Amino-beta-phenylpropionic acid**	*Unigene0101736*，*Unigene0099997*，***Unigene0050371***，*Unigene0077681*，*Unigene0069649*，	up & down
		Unigene0042451，***Unigene0013996***，*Unigene0038716*，***Unigene0037143***	down
	Hydroxybenzenecarboxylic acid		
	4-Hydroxy-3-methoxybenzaldehyde		
	**2-Coumarate**		
Histidine metabolism	**Glutamate**	*Unigene0062382*	down
	**(S)-alpha-Amino-1H-imidazole-4-propionic acid**		
Tyrosine metabolism	**2-(p-Hydroxyphenyl)ethylamine**	*Unigene0038716*，***Unigene0037143***，***Unigene0047837***	up
	**3-Methoxytyramine**		
Alanine, aspartate and glutamate	**Glutamate**	*Unigene0101443*，***Unigene0049051***，***Unigene0050969***，*Unigene0007681*，*Unigene0049135*	up & down

metabolism		*Unigene0001127*，*Unigene0045900*，*Unigene0089767*，*Unigene0028418*，*Unigene0021273*，*Unigene0089769*，***Unigene0052562***，***Unigene0045713***，***Unigene0098431***	down
Glycine, serine and threonine metabolism	**(S)-alpha-Amino-beta-(3-indolyl)-propionic acid**	*Unigene0088939*，***Unigene0019527***，*Unigene0028328*	up
	**alpha-Ketobutyrate**		
Valine, leucine and isoleucine biosynthesis	**alpha-Ketobutyrate**	*Unigene0037977*	up
		Unigene0101443，***Unigene0049051***，***Unigene0050969***，*Unigene0007681*，	up & down
Arginine biosynthesis	**Glutamate**	*Unigene0101736*，*Unigene0099997*，***Unigene0050371***，*Unigene0027433*，*Unigene0039751*	up
		Unigene0099838	down
	N2-Acetyl-L-ornithine	*Unigene0004792*	down
Phenylalanine, tyrosine and tryptophan biosynthesis	**(S)-alpha-Amino-beta-(3-indolyl)-propionic acid**	*Unigene0088939*，***Unigene0019527***，***Unigene0028328***	up & down
	**L-Phenylalanine**	*Unigene0101736*，*Unigene0099997*，***Unigene0050371***，*Unigene0077681*，*Unigene0069649*	up & down
Lysine biosynthesis	**2,6-Diaminohexanoic acid**	*Unigene0031980*	up & down
Biosynthesis of amino acids	**(S)-alpha-Amino-beta-(3-indolyl)-propionic acid**	*Unigene0088939*，***Unigene0019527***，***Unigene0028328***	up
	**2,6-Diaminohexanoic acid**	*Unigene0031980*	up

**Glutamate**	*Unigene0089767*，*Unigene0028418*，*Unigene0089769*，*Unigene0021273*，***Unigene0052562***，***Unigene0045713***，***Unigene0095431***，*Unigene0099838*	down
	Unigene0101736，*Unigene0099997*，*Unigene0039751*，***Unigene0050371***	up
**alpha-Ketobutyrate**	*Unigene0037977*，***Unigene0032304***，***Unigene0062653***，***Unigene0062655***	down
N2-Acetyl-L-ornithine	*Unigene0004792*	down
**(S)-alpha-Amino-beta-phenylpropionic acid**		

Note：Up-regulated DEGs or metabolites use bold fonts, and DEGs or metabolites down-regulated use unbolded fonts; NaCl means 200 mM NaCl treatment group; NaCl + KCl means 200 mM NaCl + 10 mM KCl treatment group.
