# Supplementary material for: Analysis of Amino Acids in the Roots of Tamarix ramosissima by Application of Exogenous Potassium (K+) under NaCl Stress
Source: Int J Mol Sci. 2022 Aug 19;23(16):9331. doi: 10.3390/ijms23169331 (PMC9409283; doi:10.3390/ijms23169331)
Supplement: Supplementary file 1 [file ijms-23-09331-s001.zip › Supplementary Table S2.pdf]

Supplementary Table S2. Analysis of differential metabolites and DEGs in 200 mM NaCl 168h vs. 200 mM NaCl NaCl + 10 mM KCl 168h amino acid-related pathways

Pathway	200 mM NaCl 168h vs. 200 mM NaCl NaCl + 10 mM KCl 168h		
	Metabolites	Genes	Regulation
Cysteine and methionine metabolism	L-Asp	*Unigene0004659*，*Unigene0004660*	down
	MTA	*Unigene0057644*，*Unigene0053473*，*Unigene0073801*<br>*Unigene0069499*	up<br>down
Tryptophan metabolism	**(S)-alpha-Amino-beta-(3-indolyl)-propionic acid**	*Unigene0017858*，*Unigene0010445*	down
	**2,3-Benzopyrrole**		
Arginine and proline metabolism	**5-Aminovaleric acid**		
	**N4-Acetylaminobutanoate**	*Unigene0095536*，*Unigene0021103*，*Unigene0021104*，*Unigene0068112*，*Unigene0053554*，*Unigene0023578*，***Unigene0051554***，***Unigene0011551***，***Unigene0015725***，***Unigene0090252***	up
Phenylalanine metabolism	**Tyrosine**		
	4-Hydroxy-3-methoxybenzaldehyde		
	**4-Hydroxycinnamate**		
	**2-Coumarate**		
Histidine metabolism	alpha-Ketoglutaric acid		
	L-Asp		
Tyrosine metabolism	**Tyrosine**	*Unigene0040692*，*Unigene0075873*，*Unigene0017858*，<br>*Unigene0049922*，***Unigene0029714***	up & down<br>down
	**2-(p-Hydroxyphenyl) ethylamine**	*Unigene0068922*，***Unigene0040077***	down
	**4-Hydroxycinnamate**		
	**3-Methoxy-4-hydroxyphenylacetate**		

	**3-Methoxytyramine**		
	3-(3,4-Dihydroxyphenyl) propanoate		
	alpha-Ketoglutaric acid	*Unigene0049051*，*Unigene0050968*，*Unigene0050969*	up & down
		Unigene0101335	up
Alanine, aspartate and glutamate metabolism	L-Asp	*Unigene0055685*，*Unigene0045213*，*Unigene0045215*，*Unigene0026789*，***Unigene0007097***	up & down
		Unigene0032025，*Unigene0037536*，*Unigene0035305*，*Unigene0073685*	down
	2-Hydroxytricarballylic acid		
	L-Asp	*Unigene0004659*，*Unigene0004660*	down
Glycine, serine and threonine metabolism	**(S)-alpha-Amino-beta-(3-indolyl)-propionic acid**	*Unigene0019527*，*Unigene0088939*，***Unigene0063642***	up
	**Bilineurine**		
	L-Asp	*Unigene0004659*，*Unigene0004660*	down
Lysine biosynthesis	alpha-Ketoglutaric acid	*Unigene0003214*，*Unigene0003215*，*Unigene0023351*	down
	**2,6-Diaminohexanoic acid**	*Unigene0101619*	up & down
Phenylalanine, tyrosine and tryptophan biosynthesis	**(S)-alpha-Amino-beta-(3-indolyl)-propionic acid**	*Unigene0019527*，*Unigene0088939*， ***Unigene0063642***	up & down
	**2,3-Benzopyrrole**	*Unigene0088939*	up
	**Tyrosine**	*Unigene0040692*，*Unigene0075873*，*Unigene0017858*	up & down
	alpha-Ketoglutaric acid	*Unigene0040692*，*Unigene0075873*，*Unigene0095229*	down
Arginine biosynthesis	L-Asp	*Unigene0035305*，*Unigene0073685*	down
	N2-Acetyl-L-ornithine	*Unigene0004792*	down

Biosynthesis of amino acids	**(S)-alpha-Amino-beta-(3-indolyl)-propionic acid**	*Unigene0019527*，*Unigene0088939*，***Unigene0063642***	up
	**Tyrosine**	*Unigene0017858*	up
	L-Asp	*Unigene0040692*，*Unigene0075873*	up
	2-Hydroxytricarballylic acid	*Unigene0004659*，*Unigene0004660*	down
		Unigene0070332，*Unigene0018830*，*Unigene0101862*，***Unigene0062635***	up
		Unigene0082111，*Unigene0066734*，*Unigene0093478*，***Unigene0077865***	down
	alpha-Ketoglutaric acid	*Unigene0095481*，*Unigene0034909*，*Unigene0017606*，*Unigene0092577*，*Unigene0010223*，***Unigene0039446***	up
		Unigene0003214，*Unigene0003215*，*Unigene0023351*	down
		Unigene0040692，*Unigene0095229*，*Unigene0075873*	up & down
	**2,6-Diaminohexanoic acid**	*Unigene0101619*	up

Note：Up-regulated DEGs or metabolites use bold fonts, and DEGs or metabolites down-regulated use unbolded fonts; NaCl means 200 mM NaCl treatment group; NaCl + KCl means 200 mM NaCl + 10 mM KCl treatment group.
