# Supplementary material for: Analysis of Amino Acids in the Roots of Tamarix ramosissima by Application of Exogenous Potassium (K+) under NaCl Stress
Source: Int J Mol Sci. 2022 Aug 19;23(16):9331. doi: 10.3390/ijms23169331 (PMC9409283; doi:10.3390/ijms23169331)
Supplement: Supplementary file 1 [file ijms-23-09331-s001.zip › Supplementary Table S3.pdf]

Supplementary Table S3. Top 20 KEGG pathways

Number	Pathway	Pathway annotated genes	Genes *P*-value	Pathway annotated metabolites	Metabolites *P*-value	Pathway ID
200 mM NaCl 48 h vs. 200 mM NaCl + 10 mM KCl 48 h						
1	Flavonoid biosynthesis	25	**0.000000**	4	0.607292	ko00941
2	Phenylpropanoid biosynthesis	63	**0.000000**	4	0.272374	ko00940
3	Zeatin biosynthesis	9	**0.004980**	1	0.658003	ko00908
4	Biotin metabolism	11	0.067159	1	0.366961	ko00780
5	Isoflavonoid biosynthesis	1	0.170712	2	0.474945	ko00943
6	Ubiquinone and other terpenoid-quinone biosynthesis	18	0.251690	1	0.785492	ko00130
7	Linoleic acid metabolism	6	0.276604	2	0.147406	ko00591
8	Pentose and glucuronate interconversions	28	0.292335	1	0.534297	ko00040
9	Glycolysis / Gluconeogenesis	106	0.349105	1	0.366961	ko00010
10	alpha-Linolenic acid metabolism	22	0.376800	1	0.456919	ko00592
11	Porphyrin and chlorophyll metabolism	22	0.392941	1	0.658003	ko00860
12	Starch and sucrose metabolism	71	0.395663	2	0.369721	ko00500
13	Indole alkaloid biosynthesis	2	0.407954	1	0.534297	ko00901
14	Amino sugar and nucleotide sugar metabolism	68	0.416889	1	0.658003	ko00520
15	Fatty acid biosynthesis	32	0.457635	1	0.534297	ko00061
16	Cysteine and methionine metabolism	71	0.512719	1	0.902307	ko00270
17	Ascorbate and aldarate metabolism	26	0.544003	3	0.119815	ko00053
18	Inositol phosphate metabolism	24	0.571471	3	**0.009604**	ko00562
19	Pentose phosphate pathway	46	0.586027	1	0.785492	ko00030
20	Glutathione metabolism	49	0.598206	2	0.257770	ko00480
200 mM NaCl 168 h vs. 200 mM NaCl + 10 mM KCl 168 h						
1	Phenylpropanoid biosynthesis	57	0.0000000	7	**0.023073**	ko00940
2	Zeatin biosynthesis	11	0.0000420	2	0.324028	ko00908
3	Flavonoid biosynthesis	12	0.0078530	3	0.887234	ko00941
4	Pentose and glucuronate interconversions	27	0.095563	2	0.191190	ko00040
5	Tryptophan metabolism	31	0.101372	2	0.510098	ko00380
6	Glycolysis / Gluconeogenesis	96	0.108210	1	0.417655	ko00010
7	Arginine and proline metabolism	37	0.112464	2	0.564616	ko00330
8	Isoflavonoid biosynthesis	1	0.142281	2	0.564616	ko00943

9	Glycerophospholipid metabolism	34	0.166781	1	0.302284	ko00564
10	Tyrosine metabolism	24	0.275695	6	**0.006492**	ko00350
11	Monobactam biosynthesis	7	0.315539	2	0.324028	ko00261
12	Glucosinolate biosynthesis	3	0.320412	2	0.191190	ko00966
13	Biotin metabolism	7	0.339885	2	0.071544	ko00780
14	Lysine degradation	23	0.344757	2	0.324028	ko00310
15	Ubiquinone and other terpenoid-quinone biosynthesis	14	0.386638	2	0.510098	ko00130
16	Cysteine and methionine metabolism	61	0.412046	2	0.738969	ko00270
17	Starch and sucrose metabolism	59	0.419110	2	0.451439	ko00500
18	Ascorbate and aldarate metabolism	23	0.422164	4	**0.043984**	ko00053
19	alpha-Linolenic acid metabolism	18	0.428574	1	0.514201	ko00592
20	Arachidonic acid metabolism	8	0.487466	1	0.888224	ko00590

Note: black bold front represents p <0.05.
