# Supplementary material for: Analysis of Amino Acids in the Roots of Tamarix ramosissima by Application of Exogenous Potassium (K+) under NaCl Stress
Source: Int J Mol Sci. 2022 Aug 19;23(16):9331. doi: 10.3390/ijms23169331 (PMC9409283; doi:10.3390/ijms23169331)
Supplement: Supplementary file 1 [file ijms-23-09331-s001.zip › Supplementary Table S4.pdf]

Supplementary Table S4. Metabolite data analysis

Name	Formula	PPM	RT [min]	m/z	Control group (mean)	Standard deviation	200 mM NaCl 48h (mean)	Standard deviation	200 mM NaCl + 10 mM KCl 48h (mean)	Standard deviation	200 mM NaCl 168h (mean)	Standard deviation	200 mM NaCl + 10 mM KCl 168h (mean)	Standard deviation
Glutamate	$C_5H_9NO_4$	2.87	1.22	146.05	164060429.27	20486907.39	80352575.07	2032000.57	131758852.07	20091973.15	115306185.87	4213705.28	107012970.71	15481353.18
5-Aminopentanoate	$C_5H_{11}NO_2$	3.94	1.45	116.07	188199312.13	41910667.26	50858588.36	6663274.74	232108313.20	23858714.81	131483707.47	6543088.36	159651755.23	14455162.27
N4-Acetylaminobutanoate	$C_6H_{11}NO_3$	2.94	1.34	144.07	448917820.33	16301180.80	422492786.57	146509665.40	597543783.73	196192005.70	911739162.10	246989126.07	2409371646.33	330914843.07

Note: the metabolites data in the non-targeted metabolome detection are relative quantitative values without units.
