# Supplementary material for: Analysis of Amino Acids in the Roots of Tamarix ramosissima by Application of Exogenous Potassium (K+) under NaCl Stress
Source: Int J Mol Sci. 2022 Aug 19;23(16):9331. doi: 10.3390/ijms23169331 (PMC9409283; doi:10.3390/ijms23169331)
Supplement: Supplementary file 1 [file ijms-23-09331-s001.zip › Supplementary Table S5.pdf]

Supplementary Table S5. DEGs and differential metabolites annotated in the arginine and proline metabolism pathway

Comparison group	Differential metabolites	Differentially expressed genes	Regulation
200 mM NaCl 48h vs. 200 mM NaCl + 10 mM KCl 48h	**Glutamate**	*Unigene0049135*	up & down
	**5-Aminopentanoate**	-	-
	-	*Unigene0033491*	-
	-	*Unigene0002920*	-
	-	*Unigene0033490*	-
	-	*Unigene0021103*	-
	-	*Unigene0068112*	-
	-	*Unigene0063796*	-
	-	*Unigene0034247*	-
	-	*Unigene0031317*	-
	-	*Unigene0089088*	-
	-	*Unigene0101525*	-
	-	*Unigene0009405*	-
	-	*Unigene0057643*	-
	-	*Unigene0073801*	-
	-	*Unigene0011304*	-
	-	*Unigene0011305*	-
	-	*Unigene0017091*	-
	-	*Unigene0101736*	-
	-	*Unigene0099997*	-
	-	***Unigene0001393***	-
	-	***Unigene0015725***	-
	-	***Unigene0040880***	-
	-	***Unigene0088054***	-
	-	***Unigene0062896***	-
	-	***Unigene0105111***	-
	-	***Unigene0073446***	-
	-	***Unigene0087849***	-
	-	***Unigene0042023***	-
	-	***Unigene0060096***	-
	-	***Unigene0104527***	-
	-	***Unigene0035833***	-
	-	***Unigene0050371***	-
200 mM NaCl 168h vs. 200 mM NaCl + 10 mM KCl 168h	**N4-Acetylaminobutanoate**	*Unigene0095536*	up
		Unigene0021103	up
		Unigene0021104	up
		Unigene0068112	up
		Unigene0053554	up
		Unigene0023578	up
		Unigene0051554	up
		Unigene0011551	up
		Unigene0015725	up
		Unigene0090252	up
	**5-Aminopentanoate**	-	-
	-	*Unigene0066232*	-
	-	*Unigene0038846*	-

	-	*Unigene0062596*	-
	-	*Unigene0076021*	-
	-	*Unigene0013401*	-
	-	*Unigene0033780*	-
	-	*Unigene0098491*	-
	-	*Unigene0044033*	-
	-	*Unigene0049135*	-
	-	*Unigene0101525*	-
	-	*Unigene0105111*	-
	-	*Unigene0098390*	-
	-	*Unigene0057644*	-
	-	*Unigene0053473*	-
	-	*Unigene0073801*	-
	-	*Unigene0075190*	-
	-	*Unigene0042023*	-
	-	*Unigene0087849*	-
	-	*Unigene0040692*	-
	-	*Unigene0075873*	-
	-	***Unigene0088054***	-
	-	***Unigene0031838***	-
	-	***Unigene0083494***	-
	-	***Unigene0049251***	-
	-	***Unigene0060096***	-
	-	***Unigene0022998***	-
	-	***Unigene0011305***	-

Note: Gene font in bold indicates gene up-regulation, gene font not bold indicates gene down-regulation; metabolite font bold indicates metabolite accumulation, metabolite font unbolded indicates metabolite degradation.
