# Supplementary material for: Analysis of Amino Acids in the Roots of Tamarix ramosissima by Application of Exogenous Potassium (K+) under NaCl Stress
Source: Int J Mol Sci. 2022 Aug 19;23(16):9331. doi: 10.3390/ijms23169331 (PMC9409283; doi:10.3390/ijms23169331)
Supplement: Supplementary file 1 [file ijms-23-09331-s001.zip › Supplementary Table S6.pdf]

Supplementary Table S6. The sequences of specific primers

ID	Primer Name	Primer Sequence (5'-3')
1	*Unigene0090252*	F: GCAGATATGAAGCATACAAGGATGAGGTT<br>R: GGAAGAATAGCAGCGGCAGTGATG
2	*Unigene0049135*	F: GTTGCGGAGAAGTTAGCTCGTGAT<br>R: GTCCACTACAGGCATAAGCATCTTGAT
3	*Unigene0021104*	F: TGCTGCTGCTCTCCACACCAA<br>R: CAGACTCCTTGAAGCCACCGAATG
4	*Unigene0068112*	F: GAAGCAACCTGAAGCCAGTGACAT<br>R: CGAGAACCAGCACAGCAGCATT
5	*Unigene0053554*	F: GTTGCTTCCCATTGTTACGGTTGAT<br>R: TCATACCACCAGCGTTGACAGTT
6	*Unigene0023578*	F: GGACAATGGTAAGCCGTATGAACAG<br>R: GGTTCGTGAAGGATCTGCGTATGA
7	*Unigene0011551*	F: AGCAACCTCAAGCCAGTGACCTT<br>R: GCGATCCAGCACAGCAGCATT
8	*Unigene0015725*	F: TCCTACTGCTGGTGCTGCTCTT<br>R: GTTGCTTCTCGCTGCCAATTCG
9	*Tubulin*	F: GCTGAGATTACAACCGCTG<br>R: CTGTTCGTTTGGTCTTGATT
